# Ultra-sensitive digital quantification of proteins and mRNA in single cells

**DOI:** 10.1038/s41467-019-11531-z

**Published:** 2019-08-07

**Authors:** Jing Lin, Christian Jordi, Minjun Son, Hoang Van Phan, Nir Drayman, Mustafa Fatih Abasiyanik, Luke Vistain, Hsiung-Lin Tu, Savaş Tay

**Affiliations:** 1 Pritzker School of Molecular Engineering, University of Chicago, Chicago, IL 60637 USA; 2 Institute for Genomics and Systems Biology, University of Chicago, Chicago, IL 60637 USA; 3 Department of Biosystems Science and Engineering, ETH Zürich, 4058 Basel, Switzerland; 4 Institute of Chemistry, Academia Sinica, Taipei, 11529 Taiwan

**Keywords:** Analytical biochemistry, Lab-on-a-chip

## Abstract

Simultaneous measurement of proteins and mRNA in single cells enables quantitative understanding and modeling of cellular functions. Here, we present an automated microfluidic system for multi-parameter and ultra-sensitive protein/mRNA measurements in single cells. Our technology improves the sensitivity of digital proximity ligation assay by up to 55-fold, with a detection limit of 2277 proteins per cell and with detection efficiency of as few as 29 protein molecules. Our measurements using this system reveal higher mRNA/protein correlation in single mammalian cells than previous estimates. Furthermore, time-lapse imaging of herpes simplex virus 1 infected epithelial cells enabled by our device shows that expression of ICP4 -a major transcription factor regulating hundreds of viral genes- is only partially correlated with viral protein counts, suggesting that many cells go through abortive infection. These results highlight the importance of high-sensitivity protein/mRNA quantification for understanding fundamental molecular mechanisms in individual cells.

## Abstract (web summary)

Digital proximity ligation assay (dPLA) can measure proteins and mRNAs in single cells, but is not compatible with cell imaging and cannot quantify rare proteins due to a high dilution factor. Here the authors present an automated microfluidic device that combines live-cell imaging, chemical stimulation, and dPLA in a smaller reaction volume.

## Introduction

Cells exhibit high degree of variability in their molecular contents^1–7^. Quantitative understanding and modeling of cellular functions require sensitive measurement of various molecular species such as messenger RNA (mRNA) and proteins. Traditionally used population-averaged techniques like Western blots and enzyme-linked immunosorbent assay (ELISA) do not capture the important differences between individual cells. Single-cell analysis is therefore necessary for an accurate understanding of cellular functions and states. As proteins directly perform many cellular functions, and mRNA levels can be poor surrogates for protein abundance in individual cells^1–3^, there is currently a tremendous need for high-sensitivity proteomic methods suitable for single-cell analysis.

Despite significant advances in single-cell analysis methods^4–7^, techniques for high-resolution protein copy number quantification are lagging behind. Current techniques such as mass spectrometry (MS) generally lack the sensitivity to detect the small amounts of proteins present in individual cells^8,9^; however, recent developments in MS are promising in detecting proteins in single cells^10^. Flow cytometry and mass cytometry (e.g., CyTOF) can detect proteins in single cells, but developing sample standards for quantification is challenging^11^. Protein immunoassays such as ELISA or enzyme-linked immunospot assay can detect proteins from single cells, but lack the sensitivity for accurate quantification. CITE-seq (cellular indexing of transcriptomes and epitopes by sequencing)^12^ and REAP-seq (RNA expression and protein sequencing)^13^ are microfluidic- and sequencing-based technologies that quantify single-cell transcriptome and epitome simultaneously in a high-throughput manner; however, they are not compatible with intracellular proteins. These methods also have limited resolution due to the high background from non-specific binding of antibodies.

To overcome those limitations, digital proximity ligation assay (dPLA) was recently introduced. dPLA provides the ability of direct and digital measurements of protein and mRNA copy numbers in single mammalian cells^2^. In dPLA, digital PCR (dPCR) is used to quantify proteins detected with a pair of oligonucleotide-tagged antibodies called PLA probes. Previously published PLA (or its close relative PEA) methods enable multiplexed simultaneous protein and mRNA measurement from single cells. Those methods claim to multiplex ~96 protein targets in a single cell; however, their quantitative polymerase chain reaction (qPCR) readout limits the sensitivity of the measurements^14–16^. The use of the dPCR readout provides significantly improved resolution and limit of detection (LOD)^17,18^, which allows direct quantification of protein copy numbers in individual mammalian cells (qPCR and dPCR comparisons are shown in Methods and Supplementary Table 1 and Supplementary Fig. 1)^2^. This advance, along with the use of dPCR for mRNA quantification, enables mathematical models of gene expression and protein translation in single mammalian cells^2^. However, currently dPLA is not able to quantify rare protein targets because of the large dilution factor introduced by sorting and lysing individual cells in micro-well plates. Further, the current dPLA method is not compatible with single-cell imaging, tracking, and chemical stimulation^2,19^. These limitations hinder many applications of regular PLA and dPLA for single-cell analysis. Genshaft et al.^20^ has presented a similar method to simultaneously profile protein (multiplexed ~27 targets) and mRNA in single cells on commercial microfluidic platforms. However, this method relies on qPCR readout and requires preamplification for both single-cell RNA and protein quantification, which can often create bias in the results and is not compatible with single-cell imaging, tracking, and chemical stimulation.

Here, we present an automated microfluidic device for dPLA measurements (µ-dPLA) that also combines live-cell imaging and chemical stimulation in the same platform. Most importantly, our system improves the detection limit and sensitivity of single-cell digital protein quantification by up to 55-fold, above an absolute limit 2277 copies of proteins per cell, and with detection efficiency (i.e., number of protein molecules converted to one DNA molecule in PLA, the detection efficiency is calculated as in Eq. (1) in Methods) of as few as 29 protein molecules, thus expanding the applicability of dPLA in single-cell studies dramatically. Our method confers the additional advantage of combining live-cell microscopy and dynamic single-cell stimulation with digital protein and mRNA measurements for multi-parametric profiling, which enables studying protein dynamics in living cells and linking it to end-point protein and mRNA measurements. Furthermore, our approach significantly reduces reagent cost and labor, and improves overall reproducibility of single-cell measurements via automated cell and reagent handling.

We applied our technology to study the correlation between protein and mRNA copy numbers in individual mammalian cells and found higher degrees of mRNA–protein correlation compared to previous estimates^2^. We showed that the ability of accurately measuring protein abundance in lowly expressing cells—which were not accounted for in previous measurements due to limited sensitivity—allow accurate characterization of mRNA-protein correlation. Finally, we used combined live-cell imaging and digital protein quantification in single cells, a new capability provided by our technology, and studied virus infection dynamics of lung epithelial cells. Live-cell imaging of herpes simplex virus 1 (HSV-1)-infected lung epithelial cells show that the expression of ICP4—a major transcription factor that regulates hundreds of viral genes—is only partially correlated with the total viral protein counts in single cells, suggesting that not all the cells infected by HSV-1 are able to go past the point of immediate-early protein synthesis and go through abortive infection. Our results highlight the importance of high-sensitivity digital measurements in understanding the relationship between gene expression and protein production in single cells in different contexts.

## Results

### Development of µ-dPLA system

In regular dPLA, cells are sorted and lysed in regular micro-wells, and then they are combined with PLA probes that bind to the target proteins. PLA probes consisting of oligonucleotide-functionalized antibodies recognize the target protein at two different epitopes, and oligonucleotides are then ligated with the help of a short piece of connector due to extreme proximity on the target protein. Droplet digital PCR (ddPCR) is used to quantify the hybridized oligonucleotides, allowing direct counting of protein copy numbers in the sample. While regular dPLA can achieve LOD at the sub-femtomolar range, the large dilution of the single-cell contents into the micro-wells results in reduced sensitivity for practical applications. Therefore, dPLA is unable to quantify protein abundances below ~30,000 copies per cell, and rarely expressed proteins are missed altogether^2^.

To improve the sensitivity of dPLA for profiling rare proteins in single cells, our approach was to sort and confine cells in nanoliter fluidic chambers at every step of the dPLA protocol. We developed an automated microfluidic device that captures and sorts individual cells into 7-nl chambers. This allowed us to dramatically reduce the dilution factor introduced from single-cell sorting and lysis steps. Furthermore, we avoided sample loss by reducing the reaction volume, so that 100% of the single-cell sample was used in the ddPCR readout. In contrast, only 9% of the sample was used in regular dPLA, which results in 91% loss of signal^2^. Furthermore, the microfluidic system performs all assay steps automatically and performs many repeated fluidic manipulations with nanoliter precision. While the typical sampling error is at ~10% when manual pipetting is used, but our automated systems reduces these pipetting errors to <0.1%, resulting in dramatically improved technical reproducibility.

**Fig. 1:**
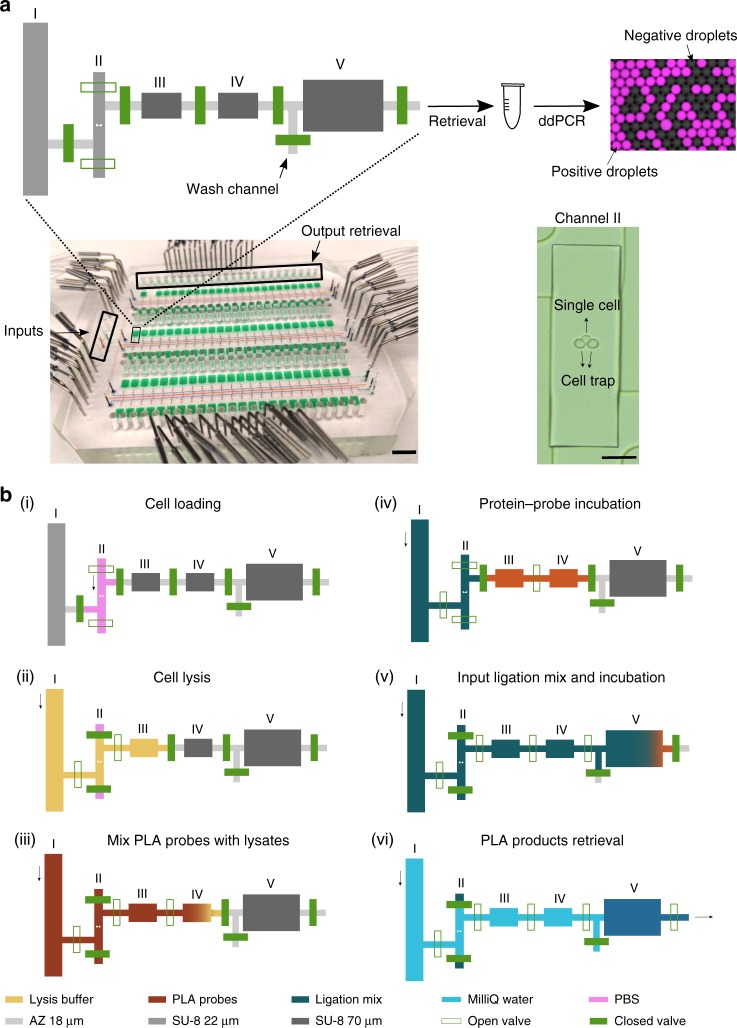
Integrated microfluidic device for performing ultrasensitive single-cell protein/messenger RNA (mRNA) measurements. **a**, Top: The schematic of one unit of assay chambers is shown; chamber sizes are not to scale. The single cell is trapped in chamber II. The stepwise assay protocol (I–V) results in a digital PCR (dPCR) readout, where counting of positive droplets allows direct quantitation of proteins or mRNA in the sample. Bottom left: The chip image with food dye loaded in different channels, the scale bar is 2 cm. Bottom right: The microscope image of a single human embryonic kidney cell trapped in chamber II, the scale bar is 50 µm. **b** Step-by-step workflow of microfluidic-digital proximity ligation assay (µ-dPLA). More information on the chip design and fabrication can be found in the Methods section

The integrated microfluidic device designed for µ-dPLA is capable of performing 144 parallel single-cell dPLA assays in an automated fashion (Fig. 1). The device traps and lyses single cells in a 7-nl chamber (Fig. 1a, chamber III). To further minimize dilution factor throughout the dPLA process, a series of microfluidic chambers were designed to precisely execute each assay step without sample loss. To avoid contamination between steps and enable automation, each chamber was separated by a set of microfluidic valves^21^, which opens specific chambers at the programmed time. Chip automation was performed by controlling valve operation using a Matlab program^19^. Measuring of precise reagents volumes was achieved by delivering reagents to empty chambers with predefined volumes, a process called dead-end filling. Wash channels were designed to prevent contamination of reagents between steps. The use of automated nanoliter fluid manipulation has the additional advantage of extremely reproducible pipetting, which reduces technical errors and variability, resulting in increased precision and sensitivity in protein and mRNA measurements.

### Ultrasensitive digital protein measurements in single cells

**Fig. 2:**
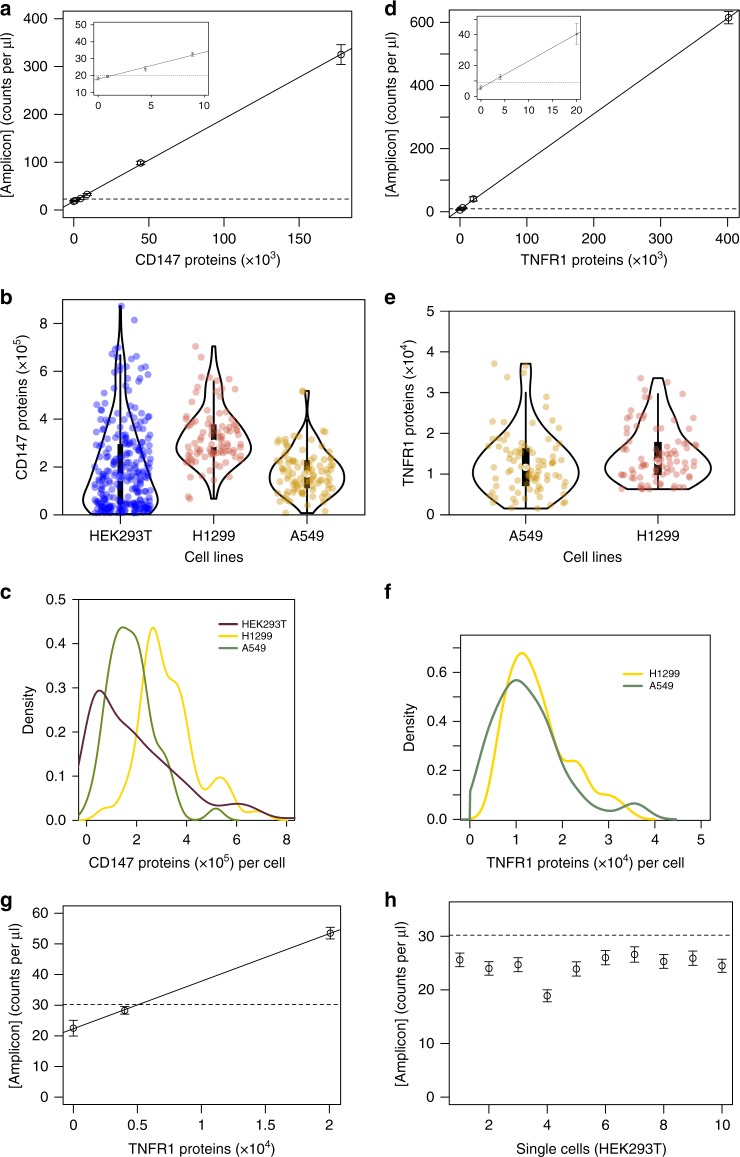
Calibration curves and single-cell protein quantification with microfluidic-digital proximity ligation assay (µ-dPLA). **a** Calibration curve for CD147 protein (inset: lower concentration region): *y* = 0.00172969*x* + 18.1043, *R*^2^ = 0.9998, dashed line indicates that limit of detection (LOD) = 2795 protein molecules. **b** Violin plots overlaid with scatter plots of single-cell CD147 counts in cell lines: human embryonic kidney cells (HEK293T), human lung epithelial cells (H1299), and human lung epithelial cells (A549). **c** Kernel density estimation of single-cell CD147 counts in the three cell lines (because kernel density estimation smooths the distribution, histograms of the single-cell data are plotted and shown in Supplementary Fig. 4). Dark purple line: HEK293T; yellow line: H1299; green line: A549. **d** Calibration curve for TNFR1 (inset: lower concentration region): *y* = 0.00151339*x* + 10.4555, *R*^2^ = 0.9992, dashed line indicates that LOD = 2277 protein molecules. **e** Violin plots overlaid with scatter plots of single-cell TNFR1 counts in cell lines: H1299 and A549. **f** Kernel density estimation of single-cell TNFR1 counts in the two cell lines (histograms of the single-cell data are plotted and shown in Supplementary Fig. 4). Yellow line: H1299; green line: A549. Inside the violin plots, the white dot of the box plot indicates the median of the data, the thick black bar in the center represents the interquartile range (25–75% data range) and the thin black line represents the 95% confidence interval. **g** On-chip calibration curve with the number of double-stranded DNA amplicons per microliter plotted against the total protein molecules in the digital PLA reaction: *y* = 0.00155149*x* + 22.3092, *R*^2^ = 0.9998, dashed line indicates the LOD, **h** the droplet digital PCR (ddPCR) readings for 10 HEK293T single cells are plotted with respect to LOD calculated from **g** shown as the dashed line, it shows all of the single-cell readings are below LOD. Error bars calculation is mentioned in the Methods section

To characterize the performance of our µ-dPLA device for protein quantification, we generated calibration curves with pure protein standards (Fig. 2a, d and Supplementary Fig. 2c). These curves show the measured number of double-stranded DNA amplicons per microliter (ddPCR readout) against the total protein molecules in chamber III (Fig. 1). Table 1 shows the LOD and detection efficiency for endogenous human proteins CD147, TNFR1, and CSTB for both on- and off-chip (regular) dPLA. We found that µ-dPLA has up to 55 times better LOD and 25 times better detection efficiency than regular dPLA. This extremely high sensitivity enables quantification of very rare protein targets: we were able to quantify 2277, 2795, and 18,824 molecules of TNFR1, CD147, and CSTB protein individual cells, respectively. Our device was able to achieve detection efficiency as low as 33, 29, and 304 copies of TNFR1, CD147, and CSTB protein molecules, respectively. Moreover, our system achieved a linear dynamic range of 3–4 orders of magnitude for these proteins. To our knowledge, this is the highest demonstrated sensitivity to date for protein quantification in single mammalian cells.

**Table 1:** Comparison of regular (off-chip) and microfluidic (on-chip) dPLA performance

	dPLA LOD—off-chip (molecules)	dPLA LOD—on-chip (molecules)	dPLA detection efficiency—off-chip (proteins/DNA)	dPLA detection efficiency—on-chip (proteins/DNA)
TNFR1	66,789^a^	2277	741^a^	33
CD147	55,385^a^	2795	70^a^	29
CSTB	1,035,991^a^	18,824^a^	5024^a^	304^a^

*dPLA* digital proximity ligation assay, *LOD* limit of detection

^a^Calibration curves are shown in Supplementary Fig. 1. Calculation of detection efficiency is explained in the Methods section

To show general applicability of our technology, we performed single-cell absolute quantification of two protein targets (CD147 and TNFR1) in three different cell types: human embryonic kidney cells (HEK293T), two human lung epithelial cell lines (H1299 and A549) (Fig. 2). A calibration curve was created along with each single-cell experiment to convert double-stranded DNA readouts to absolute protein copy numbers. Single cells were trapped and isolated on chip with an average occupancy of 90%. For measurement of the protein CD147, all of the cells captured by our device were above the LOD. Single-cell CD147 counts had an average of 197,785 proteins in HEK293T cells (*N* = 266), with a range of 3289 ± 1099 to 872,925 ± 13,393 proteins (Fig. 2b, c; defined as: average ± standard deviation, where standard deviation is calculated as in Eq. (2) in the Methods section, the standard deviation for single-cell readings is Poisson errors from ddPCR). The results generally agreed with previous reports for these cells^2^. Single-cell CD147 counts for human lung epithelial cells H1299 (*N* = 96) and A549 (*N* = 98) had averages of 327,609 and 179,513 proteins, and ranges of 67,133 ± 2476 to 704,446 ± 7428 and 7417 ± 1737 to 517,949 ± 6717 proteins, respectively (Fig. 2b, c).

About 86% of the single cells (149 out of 174) were above LOD for tumor necrosis factor receptor (TNFR1) protein measurements in our device. TNFR1 is a membrane protein and is the canonical activator of the nuclear factor-κB (NF-κB) pathway, and it has been a notoriously difficult target to quantify due to its low copy number and membrane-bound nature^22,23^. We found that single-cell TNFR1 counts had an average of 14,807 proteins in H1299 cells (*N* = 88), with a range of 6310 ± 1197 to 33,541 ± 1643 proteins (Fig. 2e, f). Single-cell TNFR1 counts in A549 cell lines (*N* = 84) had an average of 13,017 proteins, with a range of 2408 ± 927 to 37,099 ± 1382 (Fig. 2e, f; all single-cell protein raw data are provided in Supplementary Data 1). We note that single-cell TNFR1 counts were all below the LOD of dPLA (66,789 proteins, Table 1). To our knowledge, single-cell TNFR1 protein counts have not been measured before our study. This highlights the advantage of our microfluidic platform for quantification of lowly abundant proteins. Single-cell TNFR1 measurements in HEK293T cells were all below LOD of our device. Together with the finding from a live-cell imaging experiment, where HEK293T cells stimulated with high dose of TNF-α did not result in activation of the NF-κB pathway (Fig. 2g, h and Supplementary Fig. 3 and Supplementary Movie 1), the results indicate TNFR1 is either not expressed in HEK293T cells or the protein level of TNFR1 in HEK293T cells is too low to respond to TNF-α stimulation.

### Single-step joint protein/mRNA measurements in single cells

**Fig. 3:**
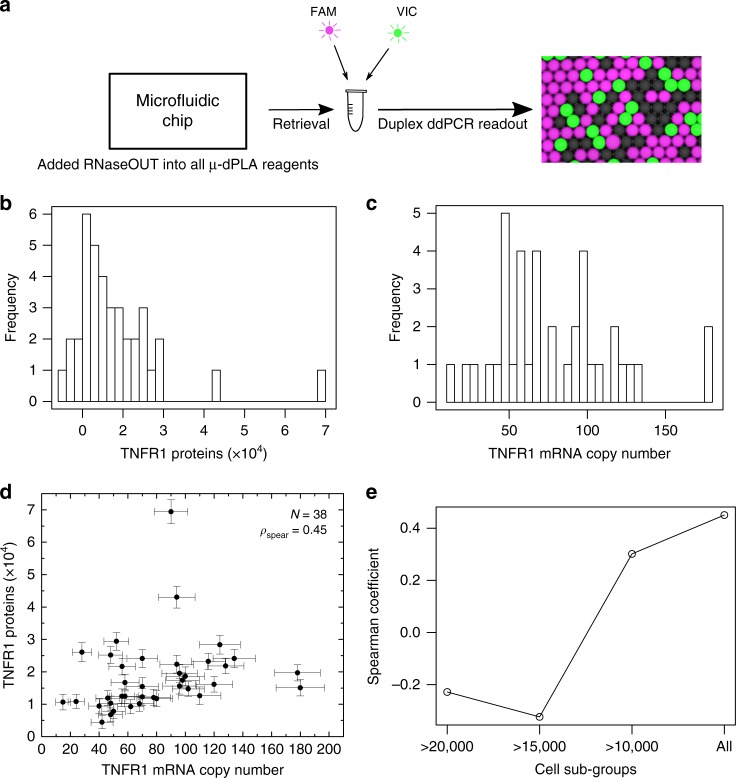
Joint digital protein/messenger RNAs (mRNAs) quantification in single cells. **a** Workflow of the duplex protein–mRNAs reaction. TaqMan probes with FAM and VIC fluorophore were used for joint quantification of protein and mRNA, respectively. **b** Histogram of TNFR1 protein counts in single human lung epithelial cells (H1299) cells. **c** Histogram of TNFR1 mRNA counts in the same single H1299 cells. **d** Joint TNFR1 mRNA and protein copy numbers in single cells is shown. See Methods for calculation of error bars. Thirty-eight single cells were measured (*N*), and the Spearman’s correlation coefficient between mRNA and protein counts is 0.45 (*ρ*). **e** The calculated Spearman’s coefficient reduces significantly if we exclude the cells that express TNFR1 at very low levels. The *X*-axis shows the threshold for exclusion, for example, >20,000 indicates that only cells with more than 20,000 TNFR1 protein copies are included in the calculation. Error bars calculation is mentioned in the Methods section

To study and model mRNA/protein expression in single cells, simultaneous measurement of protein and mRNA levels from the same single cell is needed. To achieve such joint measurements in our device, we have combined single-cell mRNA and protein quantification by integrating reverse transcription-ddPCR (RT-ddPCR) with µ-dPLA. Instead of splitting the cell lysates for protein and mRNA measurement separately, we integrated the ddPCR readout for µ-dPLA with one-step RT-ddPCR as a duplex reaction in the same reaction (Fig. 3a). This integrated protocol facilitates mRNA readout without sample bias and interference from protein measurement. TaqMan probe with FAM fluorophore was used for dPLA readout, and VIC fluorophore was used for dPCR mRNA readout. To verify whether the duplex ddPCR reaction creates crosstalk between channels, a series of control experiments were performed, which showed that VIC and FAM had negligible crosstalk (Supplementary Fig. 5a, b). Simultaneous TNFR1 protein (Fig. 3b) and mRNA (Fig. 3c) data from the same single cells (H1299) measured with the duplex reaction is shown in Fig. 3d (the calibration curve for TNFR1 with the duplex protocol is shown in Supplementary Fig. 5c). In short, we achieved simultaneous detection of proteins and mRNA in the same digital PCR droplets, which significantly simplifies the protocols for joint detection of these molecules in single cells.

### µ-dPLA shows increased single-cell mRNA–protein correlation

mRNA readouts are often used as proxy for protein abundance, but whether transcript levels accurately reflect protein levels is an open question^24^. Our high-sensitivity joint measurements reveal that the single-cell protein and mRNA correlation has a Spearman’s coefficient of 0.45 in single mammalian cells for TFNR1 expression (Fig. 3d), which is significantly higher than many previous reports^1,2,16^. We hypothesized that previous reports on low mRNA–protein correlation could be due to limited sensitivity of previous protein measurement techniques, and that sensitive quantification of lowly expressing single cells could increase the mRNA–protein correlation in the population. To test this, we excluded single cells with low protein copy numbers in our measurements and compared the changes in Spearman’s correlation coefficient (Fig. 3e). We found that the mRNA–protein correlation indeed increased as we include more cells with low protein copy numbers. This result shows the importance of high-sensitivity, multi-parameter techniques for accurate understanding of transcription and translation in single cells.

### µ-dPLA measures single-cell viral infection heterogeneity

**Fig. 4:**
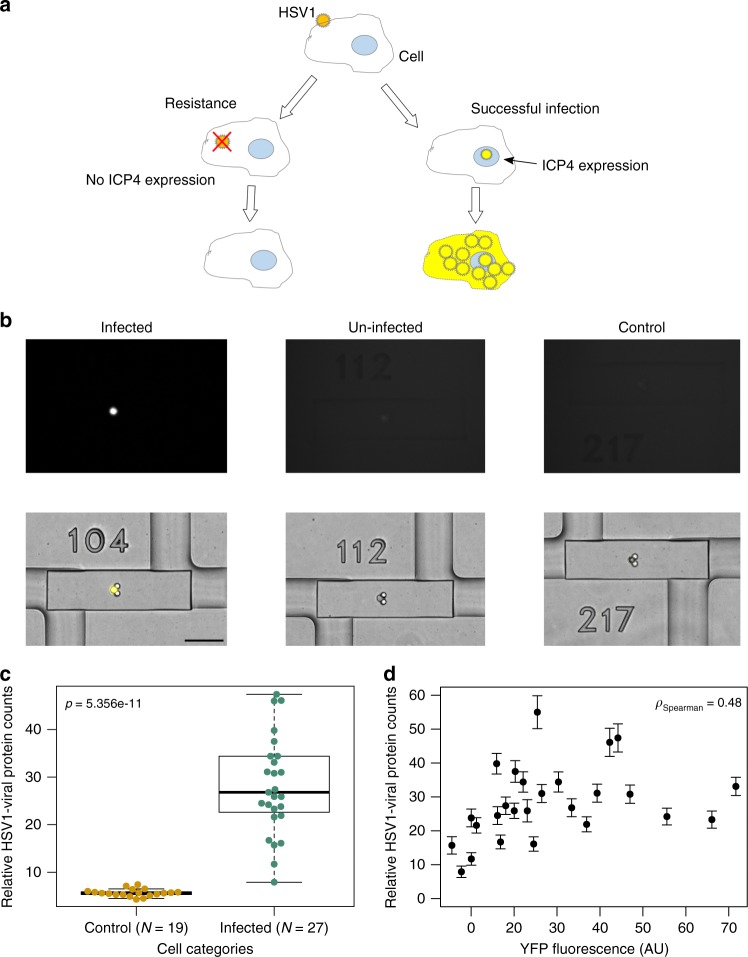
Joint live-cell imaging and digital protein analysis of HSV-1 virus infected human lung epithelial cells (A549) cells. **a** Schematic of viral infection process: if infection succeeded, viral genome goes into host cell nucleus and ICP4-YFP is expressed and viral proteins are produced; if the cell is resistant to viral infection, ICP4-YFP viral proteins will not be produced. **b** Images of ICP4-YFP (top), superimposed bright field (BF) and YFP (bottom) for infected, un-infected, and control single cells (scale bar: 100 µm). **c** Comparison of HSV-1 viral proteins counts in control cells (*N* = 19) and infected cells (*N* = 27) showed significant differences between two groups. *P* value was calculated using two-sided Welch’s *t* test. The center thick line of the box plot indicates the median of the data, the two hinges of the box represents the first and third quartile, and the whiskers indicate the full data range. **d** Correlation of the relative HSV-1 viral protein counts (microfluidic-digital proximity ligation assay (µ-dPLA) readout) with YFP fluorescence level has a Spearman’s coefficient of 0.48, which indicates that not all cells infected with HSV-1 went past the immediately-early protein synthesis stage. Error bars calculation is mentioned in the Methods section

An advantage of our µ-dPLA system is the capability of integrating live-cell microscopy for high-content single-cell profiling. To demonstrate this capability, we studied human lung epithelial cells (A549) infected with HSV-1 that expresses a yellow fluorescent protein reporter (ICP4-YFP). We infected the cells and monitored the expression of ICP4 protein in single cells by time-lapse measurements in our device. Successfully infected cells showed a nuclear YFP signal, while the un-infected cells (i.e., the cells that received the virus but infection was not successful) and control cells (the cells that received no virus) did not show any YFP expression (Fig. 4a, b). After loading the cells on chip and taking fluorescence images, all cells were lysed at the same time and processed with the µ-dPLA protocol described. We measured HSV-1 proteins with PLA probes using a polyclonal antibody that binds to ~80 viral protein species encoded by the HSV-1 genome. This allowed us to measure the abundance of ICP4 target proteins in individual cells in a single measurement. We observed a significant difference in µ-dPLA protein readout between HSV-1-infected cells (*N* *=* 27) and control cells (*N* = 19) (Fig. 4c, *p* value of 5.356e-11 calculated from two-sided Welch’s *t* test).

ICP4 is a major viral transcription factor, which is expressed immediately upon the entry of the viral genome to the host cell nucleus and prior to viral genome replication^25,26^. Thus, all YFP-positive cells in our experiments were cells in which the infection proceeded at least to the point of nuclear entry. In these cells, we measured the total viral protein content, composed of all immediate-early, early, and late proteins with µ-dPLA. Our results surprisingly showed that YFP fluorescence (ICP4 expression) is only partially correlated with the total viral protein count (Fig. 4d, Spearman’s correlation = 0.48). This suggests that not all the cells infected by HSV-1 were able to go past the point of immediate-early protein synthesis, and some of these likely represent abortively infected cells^27^ (stability of ICP4-YFP reporter was validated and shown in Supplementary Movie 2). Our method opens the door to a more in-depth analysis of this intriguing sub-population. Furthermore, our single-cell measurements of the viral proteins showed a wide distribution, with the highest expressing cells showing ~5-fold higher viral protein concentration than the lowest expressing cells. This wide distribution is in agreement with previous reports studying viral progeny release from single cells, showing substantial single-cell heterogeneity in virus infection^28,29^.

## Discussion

Here, we described an automated microfluidic system and associated protocols for ultrasensitive quantification of proteins and mRNA in individual mammalian cells. Our technique, which we called µ-dPLA, significantly improves the sensitivity for single-cell protein profiling: This method enables direct quantification of lowly abundant protein species at a limit of 2,277 protein molecules per cell, within 3 to 4 orders of dynamic range, and detection efficiency of as low as 29 protein molecules. Additional advantages of µ-dPLA compared to existing techniques include rapid processing, and more efficient use of materials and samples. A major advantage of our approach is the combined ability of cell sorting, trapping, and live-cell imaging in time-lapse experiments. With single cells trapped in isolated microfluidic chambers, µ-dPLA spares the use of fluorescence-activated cell sorting, which is bulky and expensive, and is not compatible with the study of rare cells. Additionally, µ-dPLA consumes hundreds of times less reagents and consumables compared to traditional well plate-based assays, which substantially lowers the assay cost.

Population-averaged estimates show that the cellular proteome consists of thousands of very lowly expressed proteins, with copy numbers below 10,000 molecules per cell^30^. These proteins like important regulatory transcription factors constitute an essential part of cellular proteome and hold the key to thorough understanding of cellular functions. However, existing proteomic techniques are limited in their detection limit and sensitivities, which obscures single-cell information and overlooks possible sub-populations. Our method improves the sensitivity of single-cell protein measurements dramatically, allowing the study of many important proteins that are missed with current methods.

The extremely high sensitivity provided by µ-dPLA enabled us to accurately measure the abundance of mRNA and protein molecules in a range of single cells. While earlier reports showed a poor correlation between single-cell mRNA and protein levels (Spearman’s coefficient ~ 0.1), we observed a much higher correlation between protein and mRNA levels in single mammalian cells (Spearman’s coefficient = 0.45). We have shown that high-sensitivity protein quantification plays a key role in accurately estimating protein–mRNA correlation. As more cells with lowly expressed proteins were included in the calculation, the Spearman’s coefficient increased significantly.

Most single-cell analysis techniques provide only single-modality readout, in which researchers need to choose what aspect of the single cells they want to study, such as mRNAs, proteins, or dynamics, while the rest of the cellular information is lost. In contrast, µ-dPLA confers advantages of providing multi-parametric measurement, with integration of duplex one-step RT-ddPCR reaction, live-cell microscopy, and dynamic single-cell stimulation. The integration with live-cell microscopy provides additional information on cell morphology, the expression, and translocation of fluorescent protein reporters, which is useful to decipher the relationships between different protein species or cellular pathways. We took advantage of our combined single-cell proteomic measurements with live-cell imaging and showed that HSV-1 virus infection of lung epithelial cells is highly variable. Our results surprisingly showed that the expression of ICP4, a major transcription factor that regulates hundreds of viral genes, is only partially correlated with the total viral protein counts (Spearman’s correlation = 0.48), suggesting that not all the cells infected by HSV-1 were able to go past the point of immediate-early protein synthesis and go through abortive infection. Our combined results highlight the importance of high-sensitivity protein measurements in molecular mechanisms relevant to the study of single cells.

## Methods

### Microfluidic chip fabrication

The molds were designed in AutoCAD (Autodesk, USA), and imprinted on photoresist with Heidelberg MLA 150 Direct Write Lithographer (Heidelberg Instruments Mikrotechnik GmbH, Germany). The multi-layer photoresist consists of 18 µm AZ40XT (MicroChem, USA) spun at 1976 × *g*, 22 µm SU-8 with SU-8-3025 spun at 1372 × *g*, and 70 µm two-layer SU-8 with SU-8-3025: first layer spun at 1372 × *g* and second layer spun at 448 × *g*. The AZ layer went through an overnight reflow process to obtain a rounded shape. The two-layer polydimethylsiloxane (PDMS) device used in this paper is a push-up design, which means the fluidic layer (where reagents are loaded) that is a thick layer bonds on top of the control layer (open/close valves) that is a thin layer. The molds were firstly treated with chlorotrimethylsilane (Cat. No. 92360, Sigma-Aldrich, USA) for 15 min in a fume hood. This ensured that molds were non-sticky to PDMS, and thus preserved the chip features when peeling PDMS off the molds. To cast the thick layer, 72 g of PDMS was prepared by mixing base and curing agent at 10:1 ratio. The PDMS was mixed and degassed (RTV-615, Momentive Specialty Chemicals, USA) and degassed again after poured onto the molds. To prepare a thin layer, 11 g of PDMS was prepared by mixing base and curing agent at 10:1 ratio. Then, the PDMS was spun on the mold at 448 × *g* for 1 min. Both layers were then baked at 80 °C for at least 45 min. The thick layers were peeled, punched, aligned, and bonded on top of the thin layer with oxygen plasma (input pressure 860 mTorr) for 18 s (Harrick Plasma, USA). After baking overnight at 80 °C, the fluidic-control-bonded PDMS slab was peeled and punched. The retrieval ports were punched with 1930 µm inner diameter biopsy punch (CR0950765N13R4, Syneoco, USA), and the rest of the holes were punched with 710 µm inner diameter biopsy punch (CR0350255N20R4, Syneoco, USA). Finally, the device was bonded to a glass slide (127.76 mm × 85.48 mm × 1 mm) with air plasma for 45 s (turn on the plasma at input pressure of 500 mTorr, then briefly refill the chamber with air every 10 s). The bonded device was then baked at 80 °C overnight, before it was ready to use.

### Microfluidic chip operation

The schematic of one assay unit is shown in Fig. 1. In order to block non-specific binding of reagents to PDMS surface, chips were coated with 10% bovine serum albumin (BSA) (Cat. No. 37525, Thermo Fisher, USA) for 1 h. Chips were then washed with water and ventilated by compressed air before use. The workflow of dPLA for single cells on chip is: (1) Single cells were trapped and isolated in column II. (2) lysis buffer was introduced from column I to push and lyse the single cell trapped in channel II into chamber III; lysis was completed within 10 min on ice. (3) columns I and II were washed with PLA probe before they were introduced to mix with the single-cell lysates in chamber III; the mix occupies both chamber III and IV. The chip was then placed at 37 °C for 1 h with the valve between chamber III and IV open, which allows mixing by diffusion. (4) Column I and II were washed with the ligation mix, before it was injected to push the lysate–probe mix (in chamber III and IV) to chamber V. The chip was placed on ice for 30 min for mixing by diffusion, and then placed at 37 °C for 10-min ligation and 65 °C for 5 min to heat inactivate the ligase. (5) The PLA products were flushed out by water and collected for ddPCR readout (Fig. 1b).

### Calibration curve with µ-dPLA

For all targets, a serial dilution of pure protein standards was prepared off-chip, diluted with lysis buffer (0.3× TM buffer). Lysis buffer was prepared by diluting TM buffer and protease inhibitor (Cat. No. K3011010, BioChain, USA) in cell resuspension buffer (Cat No. 4405443, Thermo Fisher, USA) to 0.3× and 1× concentration, respectively. Protein standards were loaded from the lowest to the highest concentration, with 8-min washing between samples through channel I (Fig. 1). Then, PLA probes were prepared at a concentration of 500 pM and loaded on the chip, before opening chamber IV (Fig. 1); channel I was flushed with probes for 2 min, and then channel II was flushed with probes for 15 min. The same flushing protocol also applies to the ligation mix. The rest of the protocol has been described above. After retrieving 9 µl of the final PLA products out of the chip for every unit, they were mixed with 10 µl of 2× ddPCR Supermix for Probes (Cat. no. 1863024, Bio-Rad, USA) and 1 µl of 20× Universal PCR Assay (Cat. No. 4405501, Applied Biosystems, USA). The total 20 µl of sample mix was loaded to DG8 cartridge (Cat. No. 1864008, Bio-Rad, USA) together with 65 µl of Droplet Generation Oil for Probes (Cat. No. 1863005, Bio-Rad, USA). A gasket (Cat. No. 1864007, Bio-Rad, USA) was placed on top of the cartridge holder, and then the cartridge holder was loaded into droplet generator (QX200, Bio-Rad, USA). After that, we transferred the ~40 µl droplets that were generated into a 96-well PCR plate for PCR, which was sealed with an aluminum foil. The PCR program was: 1×, 95 °C for 10 min; 40×, 94 °C for 30 s, followed by 60 °C for 1 min; 1×, 98 °C for 10 min; with the ramp speed as 1.5 °C s^−1^. The PLA probes for CD147 were conjugated with streptavidin–biotin chemistry, with commercially available oligo kit for PLA (Cat. No. 4448549, Applied Biosystems, USA) and biotinylated antibodies (CD147: BAF972; TNFR1: BAF225, R&D Systems, USA). The PLA probe conjugation protocol is provided in the kit (Cat. No. 4448549, Applied Biosystems, USA). The CSTB PLA probes were commercially available (Cat. No. 4405465, Applied Biosystems, USA).

LOD is defined as the lowest detectable number of protein molecules (calculated as 3 standard deviations of the background reading above either the background reading or the *y*-intercept of the fitted linear curve, whichever is higher). The detection efficiency is defined as the number of protein molecules converted into one DNA molecule in PLA, and it can be calculated based on the calibration curves as:

1 $${\mathrm{Detection}}\;{\mathrm{efficiency}} = \frac{{{\mathrm{Absolute}}\;{\mathrm{protein}}\;{\mathrm{copy}}\;{\mathrm{numbers}}}}{{\left[ {{\mathrm{Amplicons}}} \right] \times 20}},$$

where the ddPCR readout ([Amplicon], i.e., amplicons per µl) multiplied by 20 µl gives the total number of amplicons in the reaction.

Error bars indicate ± SD (standard deviation), and SD of the DNA concentration is calculated as^2^:

2 $${\mathrm{SD}} = \frac{{{\mathrm{CI}}_{{\mathrm{max}}} - {\mathrm{CI}}_{{\mathrm{min}}}}}{{2 \times 1.96}} \times \sqrt N.$$

Values of CI_max_ and CI_min_ can be retrieved from QuantaSoft result sheet. They are given as TotalConfMax and TotalConfMin for replicate readings, and PoissonConfMax and PoissonConfMin for single readings (such as for single-cell readings). *N* is the number of replicates. We note that the detection limit calculations shown here are based on calibration curves established using purified protein standards as is common practice, and the actual detection limit of cellular proteins might be different.

### Single-cell protein quantification with µ-dPLA

HEK293T and A549 (Cat. No. 86012804, Sigma-Aldrich, USA) cells were culture with Dulbecco’s modified Eagle’s medium (DMEM) media (Cat. No. 11965-092, Life Technologies, USA) supplemented with 10% FBS (Cat. No. F9665, Sigma-Aldrich, USA). H1299 cells were cultured with RPMI-1640 media (Cat. No. 11835-030, Life Technologies, USA) supplemented with 10% FBS. Cells cultured in flasks were dissociated with TrypLE (Cat. No. 12605028, Life Technologies, USA) for 5 min at 37 °C, and then spun down. Cells were then resuspended in sterile phosphate-buffered saline (PBS), and then filtered with a 40-µm cell strainer (Cat. No. 352340, Corning, USA). The cells were then counted and diluted to a concentration of 2 × 10^5^ per ml for on-chip single-cell loading, with an input pressure of 5 psi. After verifying on the microscope stage that cell loading was complete, the chip was immediately moved on top of ice. Single cells were lysed with 0.3× TM buffer. Lysis was complete within 10 min on ice, and was confirmed visually under a microscope. The rest of the protocol was the same as for calibration curve on chip.

### Duplex protein–mRNA digital quantification in single cells

To simultaneously quantify protein and mRNA from the same single cell, the digital PLA part of the protocol was the same, except that we added RNaseOUT (Cat. No. 10777019, Invitrogen, USA) to every reagent mix (the RNaseOUT was diluted 20 times in the final reagent mix). After retrieving 9 µl of PLA products together with mRNA from the chip, the product was mixed with 5 µl of one-step RT-ddPCR supermix, 2 µl of reverse transcriptase, 1 µl of 300 mM dithiothreitol (Cat. No. 1864021, Bio-Rad, USA), 1 µl of 20× Universal PCR assay, 1 µl of 20× TaqMan probe targeting at *TNFR1* gene (TNFR1 TaqMan Gene Expression Assay, Cat. No. Hs01042313_m1, Applied Biosystems, USA), and 1 µl of nuclease-free water. This makes up to a total of 20 µl solution. Droplets were then generated as described above, and transferred to thermal cycler for a RT-PCR program: 1×, 42 °C for 1 h, 1×, 95 °C for 10 min, 40×, 95 °C for 30 s, followed by 60 °C for 1 min, 1×, 98 °C for 10 min; with the ramp speed as 2 °C s^−1^.

### A549 infection with HSV-1 and µ-dPLA measurement

HSV-1 stock was prepared by infecting Vero cells with a multiplicity of infection (MOI) of 0.01 and harvesting 3 days later by three cycles of freezing and thawing, and the stock concertation was determined by plaque assay. A549 cells seeded in a 6-well plate were infected with 25 µl of the virus in 2 ml fresh medium overnight (MOI 2). The cells were harvested and prepared as described above to be loaded on the chip.

PLA probes for HSV-1 were conjugated in-house with the following chemistry: dibenzylcyclooctyne-PEG4-*N*-hydroxysuccinimide ester (DBCO-NHS, Sigma-Aldrich, USA) was dissolved in dimethyl sulfoxide (Sigma-Aldrich, USA) to a concentration of 3 mM. To a microcentrifuge tube was added 9 µl anti-HSV-1 antibody (Cat. No. ab9533, Abcam, USA) and 1 µl of 3 mM DBCO-NHS, mixed thoroughly, and incubated on ice for 60 min. Free DBCO-NHS was purified through a buffer exchange procedure to PBS (Life Technologies, USA) using a 50 µl, 7 K MWCO Zeba Column (Thermo Fisher, USA). Conjugation was verified by measuring absorption at 280 and 309 nm using a NanoDrop ND-1000 spectrophotometer^31^.

Probes were made by reacting the DBCO moiety on DBCO-conjugated antibodies with a terminal azide on PLA oligomers. All PLA oligomers were purchased from IDT (PLA oligo A: /5AzideN/CGCATTGCATCGTCTCGTGGGCTCGGCHHHHACHHHHACHHHNGCAGACATGCGTGATCGCTAAATCGTG; PLA oligo B: /5Phos/TCGTGTCGTGTCTAAAGTCCACATGCGTACAAAAAAAAAAAAAAAAAAAAAAAAAAAAA/3AzideN/). A solution of PBS with 0.55 mg ml^−1^ DBCO-conjugated antibody and 40 µM PLA oligomer was reacted for 72 h at 4 °C. Unreacted oligomer was removed by using a BSA Removal Kit (Abcam, USA). The final concentration of PLA probes was determined using a NanoOrange Protein Quantitation Kit (Thermo Fisher, USA). Fluorescence was measured at 485/590 nm from a 96-well black glass bottom plate (In Vitro Technologies, Australia) using an infinite M200 Pro (Tecan, Switzerland).

In all, 500 nM PLA probe stock solution was diluted to 2 nM with probe binding buffer (prepared as 0.1% BSA, 0.05% Tween-20, 100 nM goat IgG, 0.1 g l^−1^ sheared salmon sperm DNA in PBS). Then, the ligation mix was prepared by mixing 89.1 µl UltraPure water (Cat. No. 10977023, Invitrogen, USA), 10 µl of 10× ligase buffer (Cat. No. B69, Thermo Scientific, USA), 0.4 µl of 1/500 diluted T4 ligase (Cat. No. EL0011, Thermo Scientific, USA), and 0.5 µl of 1 µM of connector (TTTCGACACGACACGATTTAGGTC). Primers used for ddPCR were: GCATCGTCTCGTGGGCTC (forward) and TTGTACGCATGTGGACTTTAGACACGACACGA (reverse).

### Fluorescence calculation

Matlab function (imfindcircles) for finding circles is used to localize where the single cells are on the bright field images. First, the background was substracted from the YFP fluorescent images using ImageJ (with function Rolling Ball Background Substraction), and then the total fluorescence level was calculated by summing all the pixel values on YFP images within the circle dimensions found on bright field images.

### qPCR and ddPCR characterization

PLA experiments targeting at TNFR1 were conducted, with side-by-side readout by ddPCR (QX200, Bio-Rad) and qPCR (CFX384, Bio-Rad) for direct comparison of their performances. The same TaqMan probe was used in both PCR readouts. One TNFR1 calibration curve at concentrations: 0.5, 0.1, 0.02, 0.004 and 0.001 ng ml^−1^ was performed to determine the assay LOD with both qPCR and ddPCR. The results suggest that ddPCR produces better readings for PLA than qPCR in terms of both LOD and *R*^2^ (Supplementary Fig. 1). The LOD was calculated as background ± 3 SD. We believe the better LOD with ddPCR readout is due to smaller SDs of low concentration and buffer control readings. Then, a series of TNFR1 dilutions with concentration close to the LODs and with small concentration differences (0.012, 0.0115, 0.011, 0.0105, and 0.01 ng ml^−1^, three replicates for each concentration) were profiled, in order to compare the performances of qPCR and ddPCR at low protein/amplicon concentrations. This is relevant as the protein/amplicon concentrations are usually low for single-cell experiments and preamplification can often introduce bias in the measurements. The results show that in the low concentration range, qPCR sporadically gave no readings. Given that single-cell readings are usually low and we only have one chance to measure a single cell, we conclude that ddPCR is more sensitive and reliable for single-cell measurements. Lastly, we also conducted an experiment targeting at TNFR1 concentrations (1.3, 1.25, 1.2, 1.15, 1.1, and 1.05, 1 ng ml^−1^, three replicates for each concentration), which are well above LODs and have small concentration differences. This experiment aims to compare the performances of ddPCR and qPCR to resolve small concentration differences. Comparison results were calculated as pairwise *t* test (two-sided Welch’s *t* test) between every two concentrations and are shown in Supplementary Table 1.

### TNF-α stimulation experiment in HEK293T cells

For NF-κB activation in the HEK293T experiment, cells were seeded on an 8-well chamber slide (ibidi GmbH) coated with 0.2 mg ml^−1^ fibronectin (Merck Millipore) and cultured in DMEM high glucose (Thermo Fisher) media supplemented with 10% newborn calf serum (Thermo Fisher) and penicillin–streptomycin (Thermo Fisher). The sample was then mounted on an inverted microscope (Nikon Eclipse Ti) enclosed within a stable cell culture environment at 5% CO_2_, 95% humidity, and 37 °C (Life Imaging Services). Time-lapse live-cell images were acquired every 10 min using microscope software (NIS-Elements AR 4.20.01). For NF-κB stimulation experiment, mouse TNF-α (Gibco) was prepared and added to the sample at a final concentration of 8.3 µg ml^−1^.

### Reporting summary

Further information on research design is available in the Nature Research Reporting Summary linked to this article.

## Supplementary information

Supplementary Information

Description of Additional Supplementary Files

Supplementary Movie 1

Supplementary Movie 2

Supplementary Data 1

Reporting Summary

## Data Availability

The main datasets generated during and/or analyzed during the current study are provided in the manuscript and Supplementary information. The ddPCR raw data, HSV-1 infection images, and microfluidic chip design are available on figshare with 10.6084/m9.figshare.8289827, 10.6084/m9.figshare.8289728, 10.6084/m9.figshare.8285975, and 10.6084/m9.figshare.8290028.

## References

[1] Taniguchi Y (2010). Quantifying *E. coli* proteome and transcriptome with single-molecule sensitivity in single cells. Science.

[2] Albayrak C (2016). Digital quantification of proteins and mRNA in single mammalian cells. Mol. Cell.

[3] Greenbaum D, Colangelo C, Williams K, Gerstein M (2003). Comparing protein abundance and mRNA expression levels on a genomic scale. Genome Biol..

[4] Eilken HM, Nishikawa S-I, Schroeder T (2009). Continuous single-cell imaging of blood generation from haemogenic endothelium. Nature.

[5] Patel AP (2014). Single-cell RNA-seq highlights intratumoral heterogeneity in primary glioblastoma. Science.

[6] Young JW (2012). Measuring single-cell gene expression dynamics in bacteria using fluorescence time-lapse microscopy. Nat. Protoc..

[7] Zeisel A (2015). Cell types in the mouse cortex and hippocampus revealed by single-cell RNA-seq. Science.

[8] Aebersold R, Mann M (2003). Mass spectrometry-based proteomics. Nature.

[9] Schirle M, Heurtier M-A, Kuster B (2003). Profiling core proteomes of human cell lines by one-dimensional PAGE and liquid chromatography-tandem mass spectrometry. Mol. Cell. Proteom..

[10] Budnik B, Levy E, Harmange G, Slavov N (2018). SCoPE-MS: mass spectrometry of single mammalian cells quantifies proteome heterogeneity during cell differentiation. Genome Biol..

[11] Bendall SC (2011). Single-cell mass cytometry of differential immune and drug responses across a human hematopoietic continuum. Science.

[12] Stoeckius M (2017). Simultaneous epitope and transcriptome measurement in single cells. Nat. Methods.

[13] Peterson VM (2017). Multiplexed quantification of proteins and transcripts in single cells. Nat. Biotechnol..

[14] Fredriksson S (2002). Protein detection using proximity-dependent DNA ligation assays. Nat. Biotechnol..

[15] Gullberg M (2004). Cytokine detection by antibody-based proximity ligation. Proc. Natl. Acad. Sci. USA.

[16] Darmanis S (2016). Simultaneous multiplexed measurement of RNA and proteins in single cells. Cell Rep..

[17] Whale AS (2012). Comparison of microfluidic digital PCR and conventional quantitative PCR for measuring copy number variation. Nucleic Acids Res..

[18] Sanders R (2011). Evaluation of digital PCR for absolute DNA quantification. Anal. Chem..

[19] Kellogg RA, Gómez-Sjöberg R, Leyrat AA, Tay S (2014). High-throughput microfluidic single-cell analysis pipeline for studies of signaling dynamics. Nat. Protoc..

[20] Genshaft AS (2016). Multiplexed, targeted profiling of single-cell proteomes and transcriptomes in a single reaction. Genome Biol..

[21] Unger MA, Chou H-P, Thorsen T, Scherer A, Quake SR (2000). Monolithic microfabricated valves and pumps by multilayer soft lithography. Science.

[22] Tay S (2010). Single-cell NF-κB dynamics reveal digital activation and analogue information processing. Nature.

[23] Kellogg RA, Tay S (2015). Noise facilitates transcriptional control under dynamic inputs. Cell.

[24] Liu Y, Beyer A, Aebersold R (2016). On the dependency of cellular protein levels on mRNA abundance. Cell.

[25] DeLuca NA, McCARTHY AM, Schaffer PA (1985). Isolation and characterization of deletion mutants of herpes simplex virus type 1 in the gene encoding immediate-early regulatory protein ICP4. J. Virol..

[26] DeLuca NA, Schaffer PA (1987). Activities of herpes simplex virus type 1 (HSV-1) ICP4 genes specifying nonsense peptides. Nucleic Acids Res..

[27] Ejercito PM, Kieff E, Roizman B (1968). Characterization of herpes simplex virus strains differing in their effects on social behaviour of infected cells. J. Gen. Virol..

[28] Heldt FS, Kupke SY, Dorl S, Reichl U, Frensing T (2015). Single-cell analysis and stochastic modelling unveil large cell-to-cell variability in influenza A virus infection. Nat. Commun..

[29] Russell AB, Trapnell C, Bloom JD (2018). Extreme heterogeneity of influenza virus infection in single cells. Elife.

[30] Schwanhäusser B (2011). Global quantification of mammalian gene expression control. Nature.

[31] Gong H (2016). Simple method to prepare oligonucleotide-conjugated antibodies and its application in multiplex protein detection in single cells. Bioconjugate Chem..

