# Supplementary material for: Ultra-sensitive digital quantification of proteins and mRNA in single cells
Source: Nat Commun. 2019 Aug 7;10:3544. doi: 10.1038/s41467-019-11531-z (PMC6685952; doi:10.1038/s41467-019-11531-z)
Supplement: Supplementary file 1 — Supplementary Information [file 41467_2019_11531_MOESM1_ESM.pdf]

Supplementary Information

Ultra-Sensitive Digital Quantification of Proteins and mRNA in Single Cells

Lin, et al.

Supplementary Table 1: Two tables show *p*-values calculated from pairwise *t*-test between every 2 concentrations for qPCR (table above) and ddPCR (table below). The first row and column of both tables are TNFR1 purified protein concentrations with unit of ng ml^{-1}. The lower the *p*-values confers that the oligo concentration differences can be better resolved.

qPCR	1	1.05	1.1	1.15	1.2	1.25	1.3
1	N.A.	0.191	0.244	0.035	0.025	0.026	0.016
1.05	0.191	N.A.	0.852	0.094	0.01	0.012	0.003
1.1	0.244	0.852	N.A.	0.095	0.028	0.031	0.013
1.15	0.035	0.094	0.095	N.A.	0.427	0.499	0.084
1.2	0.025	0.01	0.028	0.427	N.A.	0.842	0.05
1.25	0.026	0.012	0.031	0.499	0.842	N.A.	0.043
1.3	0.016	0.003	0.013	0.084	0.05	0.043	N.A.

ddPCR	1	1.05	1.1	1.15	1.2	1.25	1.3
1	N.A.	0.508	0.06	0.01	0.009	0.00036	0.00051
1.05	0.508	N.A.	0.325	0.069	0.0098	0.0019	0.002
1.1	0.06	0.325	N.A.	0.054	0.03	0.0022	0.0032
1.15	0.01	0.069	0.054	N.A.	0.052	0.002	0.0031
1.2	0.009	0.0098	0.03	0.052	N.A.	0.177	0.236
1.25	0.00036	0.0019	0.0022	0.002	0.177	N.A.	0.74
1.3	0.00051	0.002	0.0032	0.0031	0.236	0.74	N.A.

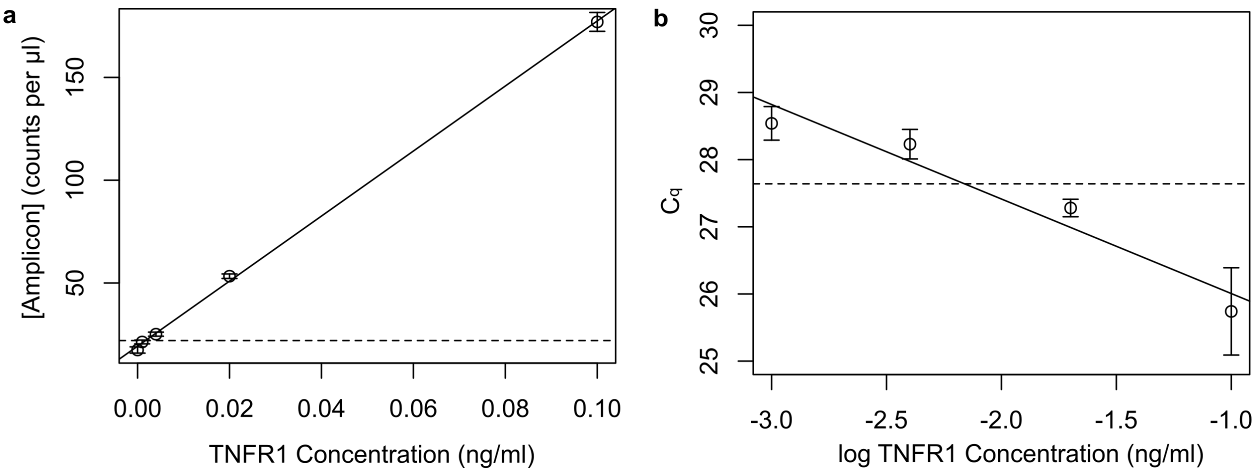

Supplementary Figure 1: Side-by-side comparison of qPCR and ddPCR with TNFR1 calibration curve. (a) TNFR1 calibration curve readout with ddPCR, $y = 19.2726 + 1581.9x$, $R^2 = 0.9995$, the dashed line indicates the LOD = 1.7 pg ml^{-1}, (b) TNFR1 calibration curve readout with qPCR, $y = 24.599 - 1.407x$, $R^2 = 0.9369$, the dashed line indicates the LOD = 6.9 pg ml^{-1}. Error bars calculation is mentioned in the Methods section.

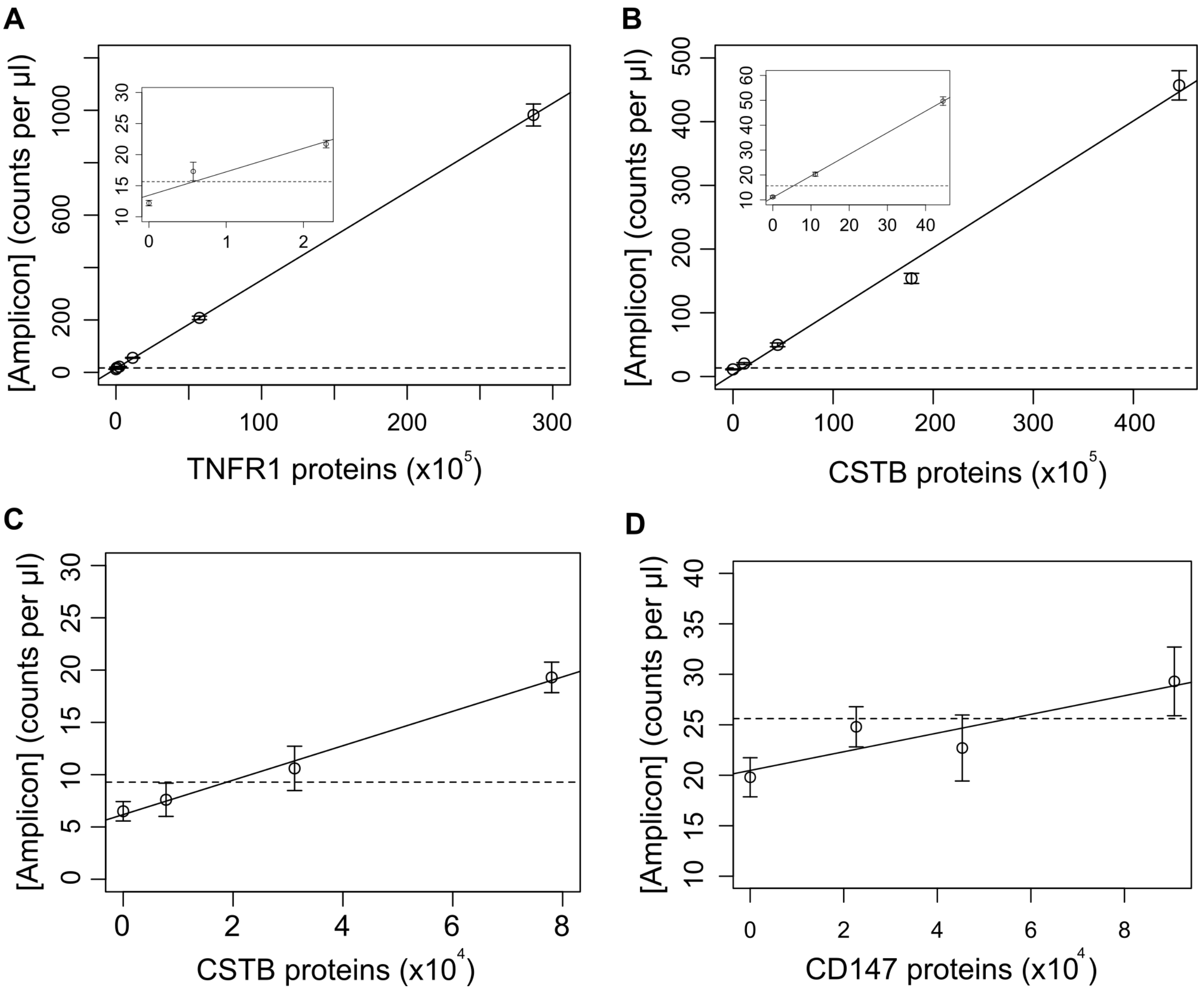

Supplementary Figure 2: Calibration curves for human TNFR1 off-chip (A), CSTB off-chip (B), CSTB on-chip (C), and CD147 off-chip (D) are plotted with number of double-stranded DNA amplicons per µl against the total protein molecules in the digital PLA reaction. Solid lines indicate linear regression for each protein and dotted lines indicate the LOD. (A) TNFR1 off-chip, $y = 3.37e\text{-}5x + 14.5146$, $R^2 = 1$, (B) CSTB off-chip, $y = 9.95e\text{-}6x + 3.14343$, $R^2 = 0.9934$, (C) CSTB on-chip, $y = 0.000164598x + 6.18522$, $R^2 = 0.9929$, (D) CD147 off-chip, $y = 0.92521x + 20.48$, $R^2 = 0.8022$. Error bars calculation is mentioned in the Methods section.

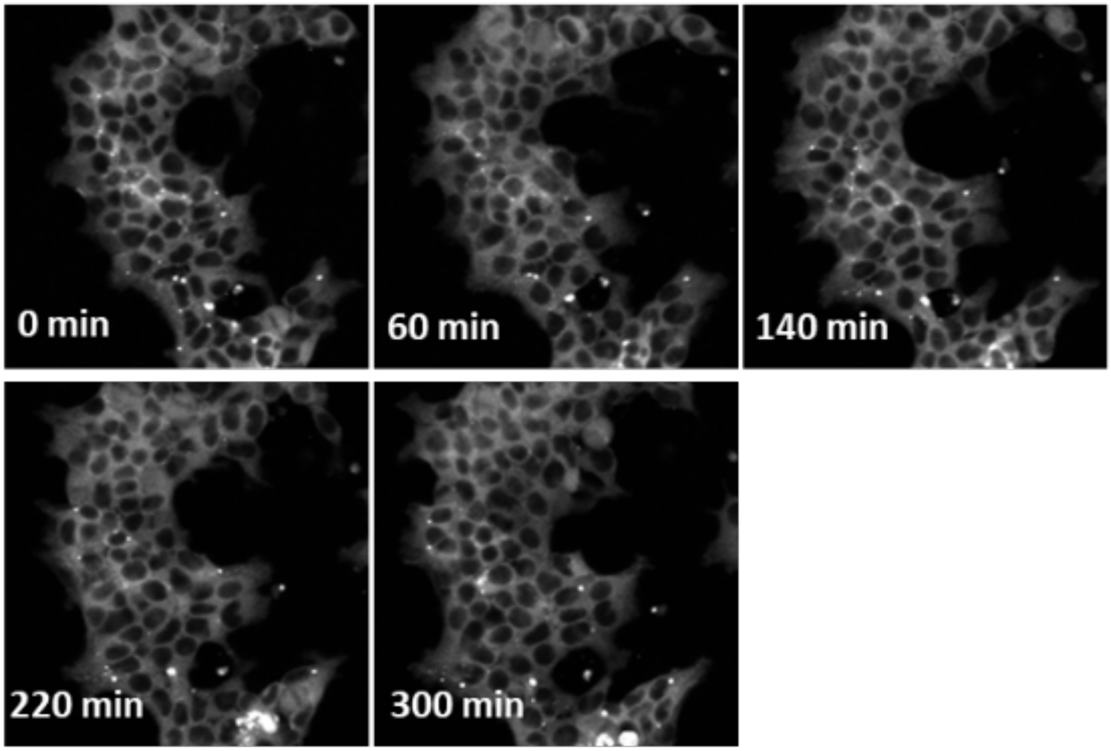

Supplementary Figure 3: p65-DsRed HEK293T cells are insensitive to TNF-α (8.3 μg ml^{-1}) stimulation, supporting the on-chip digital PLA measurement where no TNF receptor (TNFR1) was detected. Nuclear translocation of p65-DsRed are expected in stimulated cells, which is absent in the experimental observation.

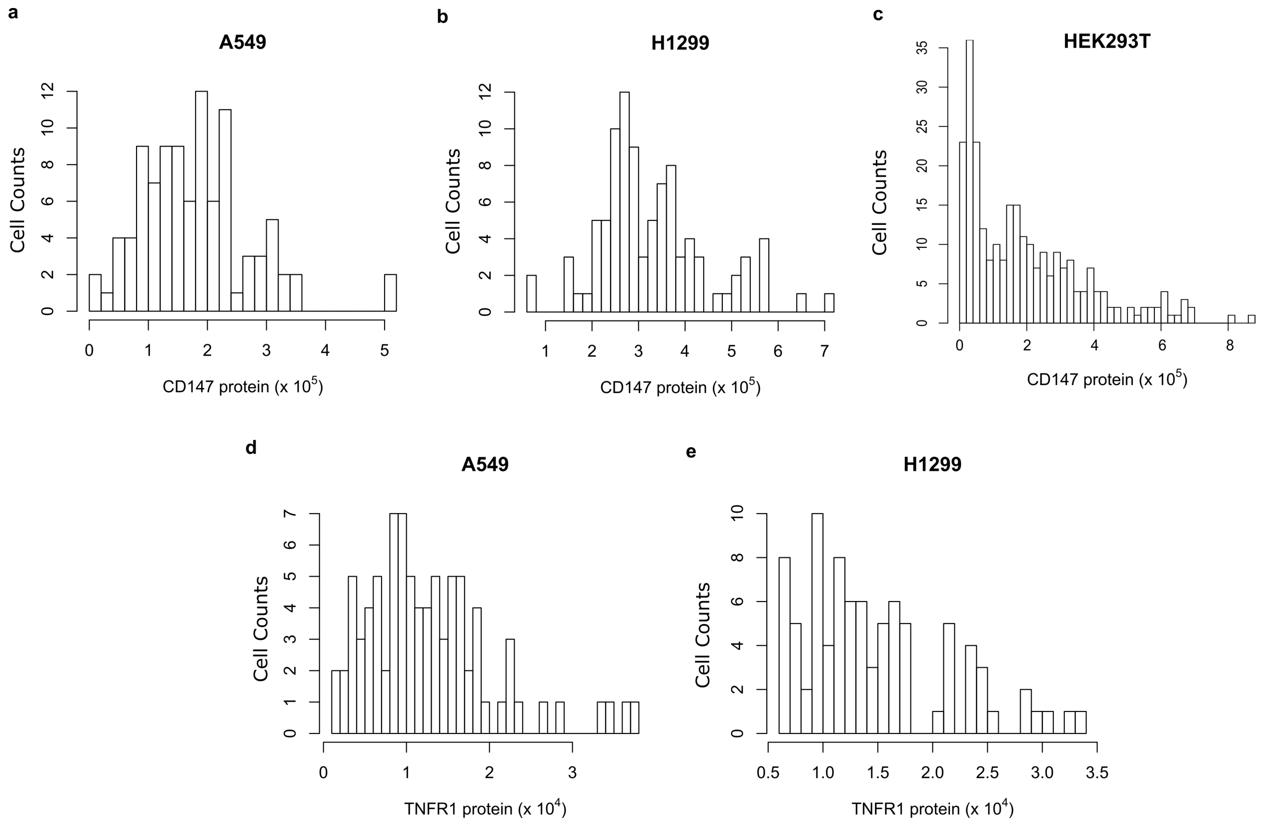

Supplementary Figure 4: Histograms of number of CD147 molecules in (a) A549, (b) H1299, (c) HEK293T single cells; and number of TNFR1 molecules in (d) A549 and (e) H1299 single cells.

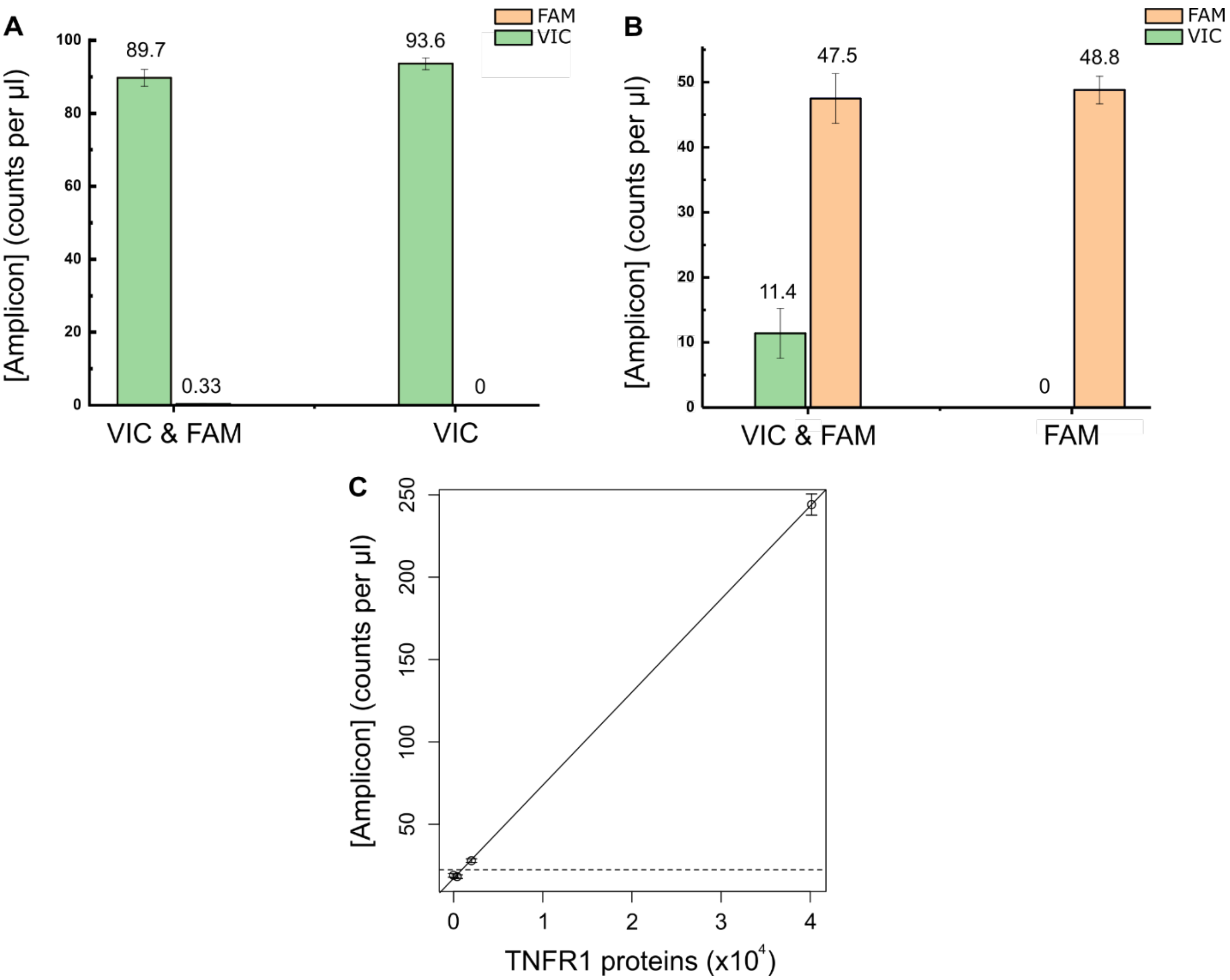

Supplementary Figure 5: Control experiments of protein-mRNA duplex reaction in bulk samples (TNFR1 probes were used), showing there is no crosstalk between FAM and VIC channels. (A) TaqMan probe with FAM fluorophore was used for protein quantification, while VIC was used for mRNA quantification. The bar on the left (VIC) shows the triplicate readings of mRNA with only VIC probe, while the ones on the right (VIC & FAM) are with both FAM and VIC probes used in one-step RT-ddPCR protocol. This verifies that with addition of FAM probe, readings in the VIC channel will be approximately the same, and VIC channel will not crosstalk to FAM channel. (B) This shows triplicate readings of both DNA products and mRNA from integrated digital PLA & one-step RT-ddPCR protocol, with only FAM probe (right, FAM) against with both FAM and VIC probes used (left, VIC & FAM). This further proves that there is no crosstalk between the two channels, and also digital PLA is compatible with one-step RT-ddPCR protocol. (C) On-chip calibration curve for TNFR1 with the duplex reaction protocol, is plotted with number of double-stranded DNA amplicons per µl against the total protein molecules in the digital PLA

reaction. Solid lines indicate linear regression for each protein and dotted lines indicate the LOD, $y = 0.000565126x + 17.1769$, $R^2 = 0.9999$. Error bars calculation is mentioned in the Methods section.
