# Supplementary material for: Ultra-sensitive digital quantification of proteins and mRNA in single cells
Source: Nat Commun. 2019 Aug 7;10:3544. doi: 10.1038/s41467-019-11531-z (PMC6685952; doi:10.1038/s41467-019-11531-z)
Supplement: Supplementary file 2 — Description of Additional Supplementary Files [file 41467_2019_11531_MOESM2_ESM.pdf]

Description of Additional Supplementary Files

File name: Supplementary Movie 1

Description: Time-lapse video showing p65-DsRed HEK293T are insensitive to high TNF-α stimulation, which supports the result of on-chip digital PLA measurement where TNF receptor (TNFR1) readings were all below limit of detection (LOD).

File name: Supplementary Movie 2

Description: Time-lapse video showing ICP4-YFP reporter signal in infected cells are stable once expressed.

File name: Supplementary Data 1

Description: Raw data of protein measurements in single cells.
