# Supplementary material for: Ultra-sensitive digital quantification of proteins and mRNA in single cells
Source: Nat Commun. 2019 Aug 7;10:3544. doi: 10.1038/s41467-019-11531-z (PMC6685952; doi:10.1038/s41467-019-11531-z)
Supplement: Supplementary file 6 — Reporting Summary [file 41467_2019_11531_MOESM6_ESM.pdf]

nature research

Corresponding author(s): savas tay 18-30233

Last updated by author(s): Jun 19, 2019

Reporting Summary

Nature Research wishes to improve the reproducibility of the work that we publish. This form provides structure for consistency and transparency in reporting. For further information on Nature Research policies, see Authors & Referees and the Editorial Policy Checklist.

Statistics

For all statistical analyses, confirm that the following items are present in the figure legend, table legend, main text, or Methods section.

n/a	Confirmed	
☐	☒	The exact sample size (*n*) for each experimental group/condition, given as a discrete number and unit of measurement
☐	☒	A statement on whether measurements were taken from distinct samples or whether the same sample was measured repeatedly
☐	☒	The statistical test(s) used AND whether they are one- or two-sided<br>*Only common tests should be described solely by name; describe more complex techniques in the Methods section.*
☒	☐	A description of all covariates tested
☒	☐	A description of any assumptions or corrections, such as tests of normality and adjustment for multiple comparisons
☐	☒	A full description of the statistical parameters including central tendency (e.g. means) or other basic estimates (e.g. regression coefficient) AND variation (e.g. standard deviation) or associated estimates of uncertainty (e.g. confidence intervals)
☐	☒	For null hypothesis testing, the test statistic (e.g. *F*, *t*, *r*) with confidence intervals, effect sizes, degrees of freedom and *P* value noted<br>*Give P values as exact values whenever suitable.*
☒	☐	For Bayesian analysis, information on the choice of priors and Markov chain Monte Carlo settings
☒	☐	For hierarchical and complex designs, identification of the appropriate level for tests and full reporting of outcomes
☒	☐	Estimates of effect sizes (e.g. Cohen's *d*, Pearson's *r*), indicating how they were calculated

Our web collection on statistics for biologists contains articles on many of the points above.

Software and code

Policy information about availability of computer code

Data collection	QuantaSoft Software from Bio-Rad (ddPCR data collection), NIS-Elements AR 5.20.00 from Nikon (microscopy data collection)
Data analysis	Customized codes written with R, python and Matlab were used for data analysis and plots

For manuscripts utilizing custom algorithms or software that are central to the research but not yet described in published literature, software must be made available to editors/reviewers. We strongly encourage code deposition in a community repository (e.g. GitHub). See the Nature Research guidelines for submitting code & software for further information.

Data

Policy information about availability of data

All manuscripts must include a data availability statement. This statement should provide the following information, where applicable:

- Accession codes, unique identifiers, or web links for publicly available datasets
- A list of figures that have associated raw data
- A description of any restrictions on data availability

The main datasets generated during and/or analyzed during the current study are provided in the manuscript and Supplementary information. The ddPCR raw data, HSV-1 infection images and microfluidic chip design are available on figshare with DOI: 10.6084/m9.figshare.8289827, 10.6084/m9.figshare.8289728, 10.6084/m9.figshare.8285975 and 10.6084/m9.figshare.8290028.

Field-specific reporting

Please select the one below that is the best fit for your research. If you are not sure, read the appropriate sections before making your selection.

☒ Life sciences ☐ Behavioural & social sciences ☐ Ecological, evolutionary & environmental sciences

For a reference copy of the document with all sections, see nature.com/documents/nr-reporting-summary-flat.pdf

Life sciences study design

All studies must disclose on these points even when the disclosure is negative.

Sample size	Sample sizes (the number of single cells analyzed in the paper) were determined by counting each single cell under microscope
Data exclusions	Data were excluded if they were from non-single cells (validated under microscope)
Replication	For calibration curves with pure protein standards, each standard concentration has at least 3 replicates from independent assay units on the same chip
Randomization	Single cells were allocated randomly
Blinding	*Describe whether the investigators were blinded to group allocation during data collection and/or analysis. If blinding was not possible, describe why OR explain why blinding was not relevant to your study.*

Reporting for specific materials, systems and methods

We require information from authors about some types of materials, experimental systems and methods used in many studies. Here, indicate whether each material, system or method listed is relevant to your study. If you are not sure if a list item applies to your research, read the appropriate section before selecting a response.

Materials & experimental systems

n/a	Involved in the study	
☐	☒	Antibodies
☐	☒	Eukaryotic cell lines
☒	☐	Palaeontology
☒	☐	Animals and other organisms
☒	☐	Human research participants
☒	☐	Clinical data

Methods

n/a	Involved in the study	
☒	☐	ChIP-seq
☒	☐	Flow cytometry
☒	☐	MRI-based neuroimaging

Antibodies

Antibodies used	anti-CD147 (R&D Systems, Cat #: BAF972), anti-TNFR1 (R&D Systems, Cat #: BAF225), anti-CSTB (Applied Biosystems, Cat #: 4405465), anti-HSV-1 (Abcam, Cat #: ab9533)
Validation	References can be found on the suppliers' websites

Eukaryotic cell lines

Policy information about cell lines

Cell line source(s)	A549 was bought from Sigma, H1299 was obtained from the lab of Prof Uri Alon, HEK293T for dPLA measurements was obtained from the lab of Prof Michela Gack, HEK293T for TNF-alpha stimulation experiment was a gift from the lab of Dr. K. Miyake.
Authentication	Cell line identity was not authenticated
Mycoplasma contamination	All cell lines in our lab are routinely monitored for mycoplasma contamination
Commonly misidentified lines (See ICLAC register)	*Name any commonly misidentified cell lines used in the study and provide a rationale for their use.*
